# Uric acid level and kidney function: a cross-sectional study of the Korean national health and nutrition examination survey (2016–2017)

**DOI:** 10.1038/s41598-020-77702-x

**Published:** 2020-12-10

**Authors:** Hye Jin Joo, Gyu Ri Kim, Dong-Woo Choi, Jae Hong Joo, Eun-Cheol Park

**Affiliations:** 1 Department of Public Health, Graduate School, Yonsei University, Seoul, Republic of Korea; 2 Institute of Health Services Research, Yonsei University, Seoul, Republic of Korea; 3 Department of Preventive Medicine and Institute of Health Services Research, Yonsei University College of Medicine, 50 Yonsei-ro, Seodaemun-gu, Seoul, 03722 Republic of Korea

**Keywords:** Diseases, Endocrinology

## Abstract

Kidney disease is expected to become the fifth leading cause of premature death globally by 2040. Uric acid level is a risk factor for kidney disease. The current study aims to investigate the association between uric acid levels and kidney function in the Korean population. The data of 11,042 participants of the 2016–2017 Korea National Health and Nutrition Examination Survey were analysed. The estimated glomerular filtration rate was calculated using the modification of diet in renal disease formula for Koreans. For each sex, uric acid levels were divided into five subsequent categories of increasing levels (Q1, Q2, Q3, Q4, and hyperuricemia). The association between uric acid level and kidney function was investigated using multiple logistic regression. The results showed that the higher the uric acid levels, the greater the odds of reduced kidney function in both sexes. In men, the adjusted odds ratios (95% confidence intervals) for reduced eGFR comparing the hyperuricemia group to the lowest serum uric acid quartile was 5.55 (3.27–9.44), and in women, the odds ratios (95% confidence intervals) was 7.52 (4.39–12.87). Normal weight or underweight in men and overweight in women, as well as diabetes mellitus, hypertension, and physical inactivity were highly associated with reduced kidney function. Our study revealed a dose–response relationship between uric acid levels and kidney function. Therefore, high uric acid level should be considered as a factor that is potentially related to kidney dysfunction in the Korean population.

## Introduction

Kidney disease is a common non-communicable condition that currently affects around 850 million people worldwide. Kidney disease is expected to become the fifth leading cause of premature deaths globally by 2040. One in 10 adults has chronic kidney disease (CKD), and every year, millions die prematurely from complications related to CKD^1,2^. CKD is a cause of substantial healthcare expenditure, and the global burden of CKD is increasing^3^. CKD requires hemodialysis or kidney transplantation, reduces quality of life, and involves a high cost for the patient^4^. Additionally, the risk of cardiovascular complications such as heart attack, stroke, and myocardial infarction is increased in CKD patients^5,6^.

The kidneys play a major role in filtering body waste and toxins from the blood and discharging them into the urine as well as in maintaining the body’s moisture, electrolyte, and acidity levels and controlling blood pressure. If these kidney functions are impaired and waste is not removed from the blood, a variety of uremic symptoms occur, some which may be life-threatening. Importantly, the kidney has a limited capacity to recover from chronic damage. The kidney performs major functions in the body, and attention should be paid to the fact that by the time symptoms manifest, kidney function is often already reduced by approximately 50%. In the face of this global public health problem, it is important to prevent the side effects of CKD by identifying and managing modifiable risk factors, such as diabetes or high blood pressure^7,8^.

As the population ages, the number of CKD patients is expected to increase at an accelerated pace, but unfortunately the lack of early diagnosis leads to the loss of opportunities for prevention^9^. The main indicator of kidney function is the blood creatinine level; creatinine accumulates in the blood, leading to elevated creatinine levels when kidney function is reduced. Kidney function can be best measured using the glomerular filtration rate (GFR), which is a measure of blood filtration by the kidneys. GFR can be easily estimated (eGFR) by measuring the blood creatinine level and considering age, ethnicity, and sex.

Diabetes, hypertension, glomerulonephritis, obesity, and aging have been consistently reported as the risk factors for CKD^10–12^. The situation with respect to uric acid level, however, is remained widely unexplored. Previously, a few studies have been conducted to investigate the relationship between serum uric acid levels and CKD suggesting that a high uric acid level could play a potential role in onset or progression of kidney disease^13–15^. According to a study examining the association between serum uric acid levels and kidney disease in Korean men, elevated serum uric acid levels were independently associated with an increased likelihood of CKD^16^. In addition, a recent study examining the association between gout and the risk of advanced CKD found that patients with gout had a higher incidence of advanced CKD than those without^17^.

Although studies on the relationship between uric acid levels and kidney function have shown controversial results for a long time, an insufficient number of studies have been performed in the Korean population. Furthermore, previous study has focused mainly on hyperuricemia^18,19^. Thus, the present study aimed to validate the association between serum uric acid level and CKD and clarify what other factors might play a potential role in developing CKD in the general South Korean population.

## Materials and methods

### Data collection and study population

Data were derived from the Korea National Health and Nutrition Examination Survey (2016–2017)^18^. KNHANES is a cross-sectional study design of nationally representative to evaluate the health and nutritional status of the Korean population aged 1 year and over. This survey is conducted annually by the Korean Centers for Disease Control and Prevention (KCDC). KNHANES employs a stratified and multistage cluster sampling design based on geographic area, gender, and age, to select household units. KNHANES contains reliable data on the general population of Korea, and is a valuable resource for development and evaluation health policies and programs in Korea.

The initial study population comprised 16,277 individuals. Among them, 16,232 subjects were selected, and those who were diagnosed with kidney failure by doctor were excluded. In addition, the subjects with missing data on uric acid levels, sex, age, socioeconomic status, or health-related factors were excluded from the study, resulting in a final sample of 11,042 individuals for analysis. KNHANES data is publicly accessible and ethical approval is not required for the use of the data. In addition, as the respondent’s information is completely anonymous, there is no need for prior consent for research purposes.

### Measurement of kidney function using the eGFR for the Korean population

The main objective of this study was assessment of kidney function. GFR is an essential aspect of kidney function evaluation. This measurement determines the level of creatinine in the blood and calculates a kidney function score that indicates how well the kidneys are functioning. Glomerular filtration rate indicates the amount of blood filtered per minute by the glomeruli. If kidney function decreases due to injury or disease, the filtration rate decreases, and waste products begin to accumulate in the blood. Clinically, the eGFR is mainly used to detect kidney status. eGFR is calculated on the basis of a serum creatinine test. Creatinine is a muscle waste product that is filtered out of the blood by the kidneys and is released into the urine at a relatively constant rate.

The eGFR was calculated using the modification of diet in renal disease (MDRD) formula. In 2010, a Korean MDRD formula was developed, which was reported to measure the glomerular filtration rate of Koreans more accurately than the conventional MDRD formula. The Korean MDRD formula for the eGFR is as follows: 107.904 × (Creatinine in mg/dL)^−1.009^ × (age)^−0.02^ × (0.667 if female)^19^. Therefore, in this study, kidney function was confirmed using the Korean MDRD formula. The normal range of glomerular filtration rate is about 90–120 mL per minute. A reduction of the GFR can be interpreted as impaired kidney function. According to the eGFR formula, participants with a GFR of less than 90 mL were considered to have reduced kidney function. In order to identify malfunction of kidney function, the dependent variable was designed in the form of a binary division with a cut-off of 90 mL (i.e., decreased function group: < 90 mL/min/1.73 m^2^, normal group: ≥ 90 mL/min/1.73 m^2^)^2^.

### Uric acid levels

The main exposure of interest was serum uric acid levels (SUA). Venous blood samples were collected in the morning after an overnight fast. In the KNHANES, SUA was measured by means of a Hitachi automatic analyzer 7600-210 using a colorimetric enzymatic method. SUA levels were then divided into five categories in both sexes: one group for hyperuricemia (> 7.00 mg/dL for men; > 6.00 mg/dL for women^20,21^), and four groups (Q1, Q2, Q3, and Q4) within the reference range according to sex-specific quartiles. The SUA quartiles were as follows: < 4.80 mg/dL, 4.80–5.49 mg/dL, 5.50–6.09 mg/dL, 6.10–6.99 mg/dL for men; and < 3.70 mg/dL, 3.70–4.19 mg/dL, 4.20–4.79 mg/dL, 4.80–5.99 mg/dL for women. The groups under 4.80 mg/dL (men) and 3.70 mg/dL (women) were set as the reference groups.

### Covariates

The following covariates were included in the fully adjusted models because they could be associated with kidney function and uric acid level: age (< 40, 40–49, 50–59, 60–69, ≥ 70), household income group (low, medium–low, medium–high, or high), educational level (high school or under, university or above), region (metropolitan or rural), and occupational categories (white collar, pink collar, blue collar, or unemployed). Household income groups classified as quartiles were calculated by dividing household income by the square root of the number of household members, a standard method recommended by the Organisation for Economic Cooperation and Development^22^. Occupations were categorised according to the Korean version of the Standard Classification of Occupations based on the International Standard Classification of Occupations by the International Labor Organization^23^. We restructured the classification into four categories: white (office work), pink (sales and service), blue (agriculture, forestry, fishery, and armed forces occupations), and unemployed.

Health-related covariates included body mass index (normal or underweight, overweight, or obese), waist circumference (normal circumference or abdominal obesity), diabetes mellitus, hypertension, dyslipidaemia, smoking status (non-smoker, ex-smoker, or current smoker), frequency of drinking (never, occasionally, or frequently), physical activity (active or inactive). Waist circumference was divided using the Korean abdominal obesity criteria, which is 90 cm for men and 85 cm for women^24^. The diagnoses of diabetes mellitus, hypertension, and dyslipidaemia were confirmed by an experienced physician. Physical activity was assessed by the ability of the subject to engage in a moderate-intensity activity for more than 150 min, a high intensity activity for more than 75 min, or a combination of both moderate and high intensity-activity (1 min of high intensity and 2 min of moderate intensity) per week^25^.

### Statistical analysis

Owing to the considerable sex differences in physical functions such as the capacity of female hormones to effectively reduce uric acid levels, all analyses have been stratified by sex. Sampling weights were applied in all data analyses to perform multistage stratified probability sampling of KNHANES. Descriptive analysis was performed to examine the distribution of the general characteristics of the study population. We calculated the frequency and percentages for each variable through the chi-square test. The statistical significance level was set as p-value of lower than 0.05. To identify the association between uric acid levels and kidney function, a multiple logistic regression analysis was performed after adjusting sociodemographic and health-related covariates. Odds ratios (ORs) and 95% confidence intervals (CIs) were calculated in order to perform comparisons between subjects with uric acid levels below 4.80 mg/dL (males) or 3.70 mg/dL (females) and subjects with different levels of uric acid (males: 4.80–5.49 mg/dL, 5.50–6.09 mg/dL, 6.10–6.99 mg/dL, over 7.00 mg/dL; females: 3.70–4.19 mg/dL, 4.20–4.79 mg/dL, 4.80–5.99 mg/dL, over 6.00 mg/dL). Subgroup analysis was performed by body mass index (BMI), waist circumference, age, previously diagnosed diabetes, previously diagnosed hypertension, previously diagnosed dyslipidaemia. In addition, multicollinearity was tested using the variance inflation factors. All statistical analyses were performed using SAS 9.4 software (SAS Institute, Cary, NC, USA).

## Results

Table 1 presents the general characteristics of the gender-stratified study population. Overall, 922 (19.0%) of the 4,848 men and 403 (6.5%) of the 6194 women included in the study were considered hyperuricemic respectively. In general, among both men and women, higher uric acid levels were associated with lower kidney function (*p* < 0.001 for both men and women). Specifically, the percentages of decreased kidney function among men were 6.6%, 9.3%, 10.8%, 15.4%, and 24.8% in the < 4.80, 4.80–5.49, 5.50–6.09, 6.10–6.99, and ≥ 7.00 mg/dL groups, respectively, while among women, the proportions of decreased kidney function were 19.8, 25.9, 35.5, 43.1, and 61.8% in the < 3.70, 3.70–4.19, 4.20–4.79, 4.80–5.99, and ≥ 6.00 mg/dL groups, respectively.

**Table 1:** General characteristics of the study population.

	Kidney function: eGFR (mL/min per 1.73 m^2^)ª													
	Men (n = 4848)							Women (n = 6194)						
	TOTAL		Decreased function^c^		Normal function^d^		*P* value	TOTAL		Decreased function		Normal function		*P* value
	N	%	N	%	N	%		N	%	N	%	N	%	
**Uric acid level (mg/dL)**^**b**^							< 0.0001							< 0.0001
Q1	951	19.6	63	6.6	888	93.4		1367	22.1	270	19.8	1097	80.2	
Q2	949	19.6	88	9.3	861	90.7		1318	21.3	341	25.9	977	74.1	
Q3	945	19.5	102	10.8	843	89.2		1506	24.3	535	35.5	971	64.5	
Q4	1081	22.3	166	15.4	915	84.6		1600	25.8	689	43.1	911	56.9	
Hyperuricemia	922	19.0	229	24.8	693	75.2		403	6.5	249	61.8	154	38.2	
**BMI**^**e**^							< 0.0001							0.001
Underweight or Normal (< 23.0)	1610	33.2	166	10.3	1444	89.7		3031	48.9	950	31.3	2081	68.7	
Overweight (23.0–24.9)	1267	26.1	173	13.7	1094	86.3		1267	20.5	456	36.0	811	64.0	
Obese (≥ 25.0)	1971	40.7	309	15.7	1662	84.3		1896	30.6	678	35.8	1218	64.2	
**Waist circumference**							< 0.0001							
Normal	2133	44.0	226	10.6	1907	89.4		4457	72.0	1430	32.1	3027	67.9	
Abdominal obesity	2715	56.0	422	15.5	2293	84.5		1737	28.0	654	37.7	1083	62.3	
**Age**							< 0.0001							< 0.0001
< 40	1403	28.9	116	8.3	1287	91.7		1739	28.1	426	24.5	1313	75.5	
40–49	911	18.8	95	10.4	816	89.6		1175	19.0	346	29.4	829	70.6	
50–59	906	18.7	85	9.4	821	90.6		1206	19.5	378	31.3	828	68.7	
60–69	842	17.4	133	15.8	709	84.2		1072	17.3	422	39.4	650	60.6	
≥ 70	786	16.2	219	27.9	567	72.1		1002	16.2	512	51.1	490	48.9	
**Household income**							< 0.0001							< 0.0001
Low	828	17.1	166	20.0	662	80.0		1233	19.9	515	41.8	718	58.2	
Mid-low	1157	23.9	154	13.3	1003	86.7		1523	24.6	491	32.2	1032	67.8	
Mid-high	1373	28.3	165	12.0	1208	88.0		1699	27.4	501	29.5	1198	70.5	
High	1490	30.7	163	10.9	1327	89.1		1739	28.1	577	33.2	1162	66.8	
**Educational level**							0.0002							< 0.0001
≤ High school	2394	49.4	364	15.2	2030	84.8		3704	59.8	1353	36.5	2351	63.5	
≥ College	2454	50.6	284	11.6	2170	88.4		2490	40.2	731	29.4	1759	70.6	
**Region**							0.278							0.012
Metropolitan	2138	44.1	273	12.8	1865	87.2		2725	44.0	963	35.3	1762	64.7	
Rural	2710	55.9	375	13.8	2335	86.2		3469	56.0	1121	32.3	2348	67.7	
**Occupational categories**^**f**^							< 0.0001							< 0.0001
White	1417	29.2	159	11.2	1258	88.8		1346	21.7	410	30.5	936	69.5	
Pink	483	10.0	30	6.2	453	93.8		890	14.4	251	28.2	639	71.8	
Blue	1620	33.4	199	12.3	1421	87.7		960	15.5	305	31.8	655	68.2	
Unemployed	1328	27.4	260	19.6	1068	80.4		2998	48.4	1118	37.3	1880	62.7	
**Diabetes mellitus**							< 0.0001							< 0.0001
Yes	513	10.6	121	23.6	392	76.4		539	8.7	262	48.6	277	51.4	
No	4335	89.4	527	12.2	3808	87.8		5655	91.3	1822	32.2	3833	67.8	
**Hypertension**							< 0.0001							< 0.0001
Yes	1278	26.4	307	24.0	971	76.0		1374	22.2	648	47.2	726	52.8	
No	3570	73.6	341	9.6	3229	90.4		4820	77.8	1436	29.8	3384	70.2	
**Dyslipidemia**							< 0.0001							< 0.0001
Yes	757	15.6	143	18.9	614	81.1		1215	19.6	509	41.9	706	58.1	
No	4091	84.4	505	12.3	3586	87.7		4979	80.4	1575	31.6	3404	68.4	
**Smoking status**							< 0.0001							0.316
Current smoker	1712	35.3	178	10.4	1534	89.6		303	4.9	90	29.7	213	70.3	
Ex-smoker	2004	41.3	326	16.3	1678	83.7		353	5.7	122	34.6	231	65.4	
Non-smoker	1132	23.3	144	12.7	988	87.3		5538	89.4	1872	33.8	3666	66.2	
**Drinking status**							< 0.0001							< 0.0001
Frequently	1772	36.6	204	11.5	1568	88.5		708	11.4	184	26.0	524	74.0	
Occasionally	2256	46.5	286	12.7	1970	87.3		3350	54.1	1077	32.1	2273	67.9	
Never	820	16.9	158	19.3	662	80.7		2136	34.5	823	38.5	1313	61.5	
**Physical activity**							0.044							0.071
Active	2288	47.2	282	12.3	2006	87.7		2589	41.8	838	32.4	1751	67.6	
Inactive	2560	52.8	366	14.3	2194	85.7		3605	58.2	1246	34.6	2359	65.4	
**Year**							0.335							0.014
2016	2391	49.3	331	13.8	2060	86.2		3136	50.6	1074	34.2	2062	65.8	
2017	2457	50.7	317	12.9	2140	87.1		3058	49.4	1010	33.0	2048	67.0	
**Total**	4848	100.0	648	13.4	4200	86.6		6194	100.0	2084	33.6	4110	66.4	

ªGlomerular filtration rate for the Korean population, estimated using the Modification of Diet in Renal Disease equation (mL/min per 1.73 m^2^) = 107.904 × (Creatinine in mg/dL)^−1.009^ × (age)^−0.02^ × 0.667 [if female].

^b^Q1: < 4.80 mg/dL, < 3.70 mg/dL; Q2: 4.80–5.49 mg/dL, 3.70–4.19 mg/dL; Q3: 5.50–6.09 mg/dL, 4.20–4.79 mg/dL; Q4: 6.10–6.99 mg/dL, 4.80–5.99 mg/dL; Hyperuricemia: ≥ 7.00 mg/dL, ≥ 6.00 mg/dL, respectively.

^c^Decreased function = eGFR < 90 mL/min per 1.73 m^2^; ^d^Normal function = eGFR 90 ≥ mL/min per 1.73 m^2^.

^e^BMI body mass index; Obesity status defined by BMI based on the 2018 Clinical Practice Guidelines for Overweight and Obesity in Korea.

^f^Three groups (white, pink, and blue) based on the International Standard Classification of Occupations Codes. Unemployed group includes housewives.

Table 2 shows the logistic regression analysis results for both men and women, adjusted for all covariates. We observed a dose–response relationship between uric acid levels and kidney function in both men and women. In particular, compared with the reference group, the odds ratios (95% CIs) for kidney dysfunction in men were as follows: OR = 1.49 [95% CI 0.92–2.39] for 4.80–5.49 mg/dL; OR = 1.70 [95% CI 1.10–2.64] for 5.50–6.09 mg/dL; OR = 3.02 [95% CI 1.96–4.67] for 6.10–6.99 mg/dL; OR = 5.49 [95% CI 3.64–8.29] for ≥ 7.00 mg/dL. Among women, the odds ratios (95% Cis) across the uric acid level groups in women were as follows: OR = 1.41 [95% CI 1.12–1.77] for 3.70–4.19 mg/dL; OR = 2.24 [95% CI 1.82–2.76] for 4.20–4.79 mg/dL; OR = 3.12 [95% CI 2.52–3.86] for 4.80–5.99 mg/dL; OR = 5.79 [95% CI 4.37–7.66] for ≥ 6.00 mg/dL. In other words, the higher the uric acid levels, the higher the odds ratios for reduced eGFR. The odds ratio was significant in all groups for both sexes except for men in the 4.80–5.49 mg/dL group. The hyperuricemia groups in both genders showed the highest risk of kidney function reduction.

**Table 2:** Odds ratio for decreased kidney function.

Variables	Kidney function: eGFR (mL/min per 1.73 m^2^)ª			
	Men		Women	
	Adjusted OR^b^	95% CI	Adjusted OR^b^	95% CI
**Uric acid level (mg/dL)**^**c**^				
Q1	1.00		1.00	
Q2	1.49	(0.92–2.39)	1.41	(1.12–1.77)
Q3	1.70	(1.10–2.64)	2.24	(1.82–2.76)
Q4	3.02	(1.96–4.67)	3.12	(2.52–3.86)
Hyperuricemia	5.49	(3.64–8.29)	5.79	(4.37–7.66)
**BMI**^**d**^				
Normal or underweight (< 23.0)	1.00		1.00	
Overweight (23.0–24.9)	1.45	(1.05–2.00)	0.87	(0.73–1.04)
Obese (≥ 25.0)	1.61	(1.17–2.22)	0.74	(0.60–0.90)
**Waist circumference**				
Normal	1.00		1.00	
Abdominal obesity	0.80	(0.60–1.05)	0.90	(0.73–1.11)
**Age**				
< 40	1.00		1.00	
40–49	1.58	(1.15–2.18)	1.42	(1.17–1.72)
50–59	1.36	(0.93–1.98)	1.61	(1.31–1.98)
60–69	2.30	(1.55–3.43)	2.24	(1.75–2.88)
≥ 70	4.63	(3.11–6.90)	3.14	(2.37–4.17)
**Household income**				
Low	1.00		1.00	
Mid-low	0.94	(0.66–1.34)	0.91	(0.74–1.12)
Mid-high	0.92	(0.66–1.29)	0.93	(0.75–1.15)
High	0.77	(0.55–1.08)	1.09	(0.87–1.36)
**Educational level**				
≤ Highschool	1.00		1.00	
≥ College	1.37	(1.04–1.79)	1.05	(0.88–1.25)
**Region**				
Metropolitan	1.00		1.00	
Rural	1.04	(0.84–1.29)	0.80	(0.70–0.92)
**Occupational categories**^**e**^				
White	1.05	(0.77–1.44)	1.06	(0.87–1.28)
Pink	0.52	(0.33–0.81)	0.81	(0.66–0.99)
Blue	0.87	(0.66–1.14)	0.89	(0.73–1.09)
Unemployed	1.00		1.00	
**Diabetes**				
Yes	1.30	(0.98–1.74)	1.38	(1.08–1.77)
No	1.00		1.00	
**Hypertension**				
Yes	2.19	(1.70–2.82)	1.28	(1.04–1.56)
No	1.00		1.00	
**Dyslipidemia**				
Yes	0.92	(0.70–1.23)	0.96	(0.80–1.14)
No	1.00		1.00	
**Smoking status**				
Current smoker	1.03	(0.78–1.37)	1.37	(1.03–1.83)
Ex-smoker	0.93	(0.69–1.25)	0.92	(0.69–1.24)
Non-smoker	1.00		1.00	
**Drinking status**				
Frequently	0.91	(0.70–1.18)	0.99	(0.86–1.14)
Occasionally	0.67	(0.49–0.92)	0.83	(0.64–1.08)
Never	1.00		1.00	
**Physical activity**				
Active	1.00		1.00	
Inactive	0.96	(0.77–1.18)	0.97	(0.84–1.10)
**Year**				
2016	1.04	(0.85–1.28)	1.08	(0.94–1.25)
2017	1.00		1.00	

ªModification of Diet in Renal Disease estimated glomerular filtration rate for Korean population (mL/min per 1.73 m^2^) = 107.904 × (Creatinine in mg/dL)^−1.009^ × (age)^−0.02^ × 0.667 [if female].

^b^OR adjusted for all sociodemographic, economic, health-related factors considered in the study.

Q1: < 4.80 mg/dL, < 3.70 mg/dL; Q2: 4.80–5.49 mg/dL, 3.70–4.19 mg/dL; Q3: 5.50–6.09 mg/dL, 4.20–4.79 mg/dL; Q4: 6.10–6.99 mg/dL, 4.80–5.99 mg/dL; Hyperuricemia: ≥ 7.00 mg/dL, ≥ 6.00 mg/dL, respectively.

^d^BMI body mass index; Obesity status defined by BMI based on 2018 Clinical Practice Guidelines for Overweight and Obesity in Korea.

^e^Three groups (white, pink, blue) based on the International Standard Classification Occupations codes. Unemployed group includes housewives.

**Figure 1:**
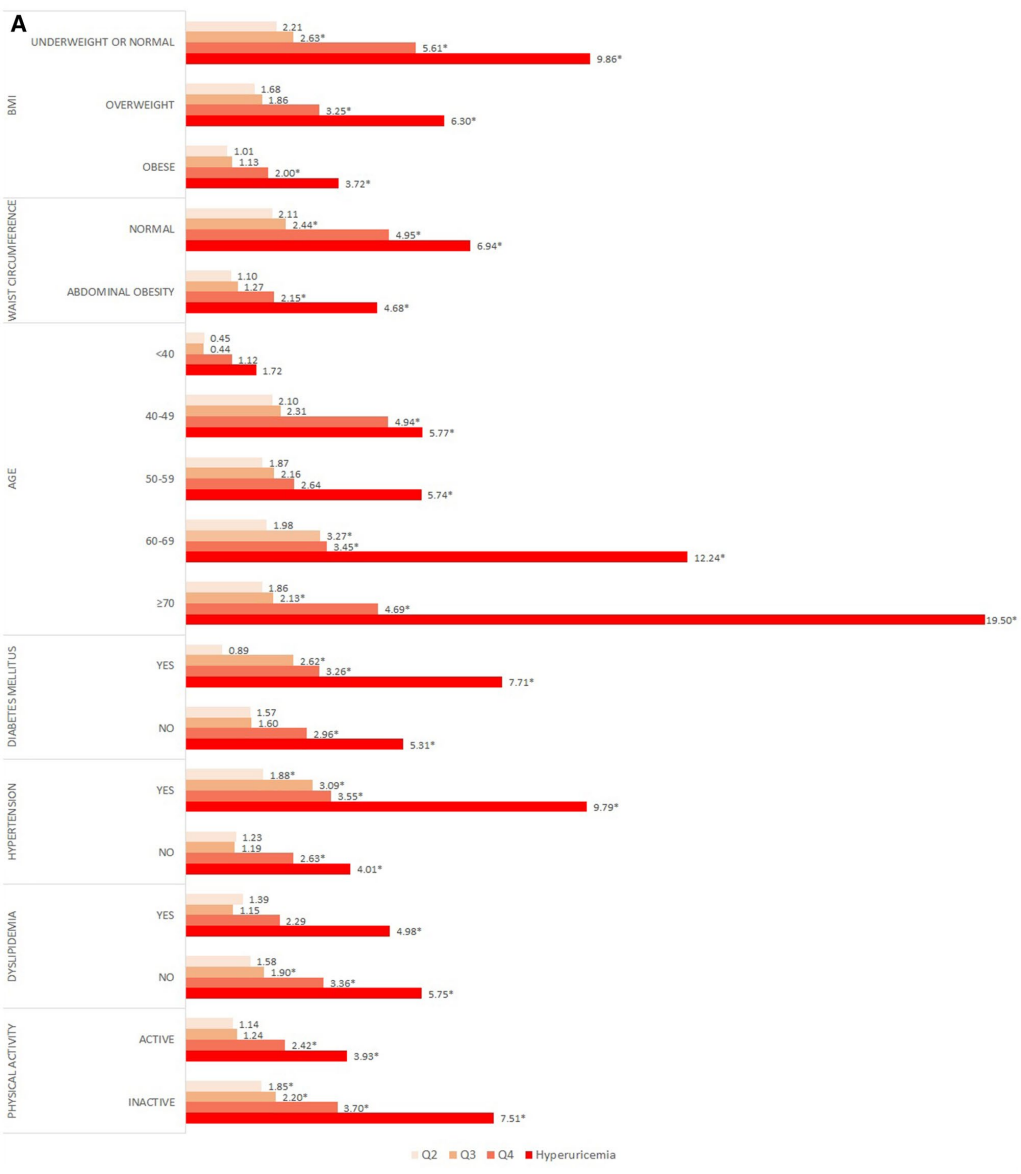

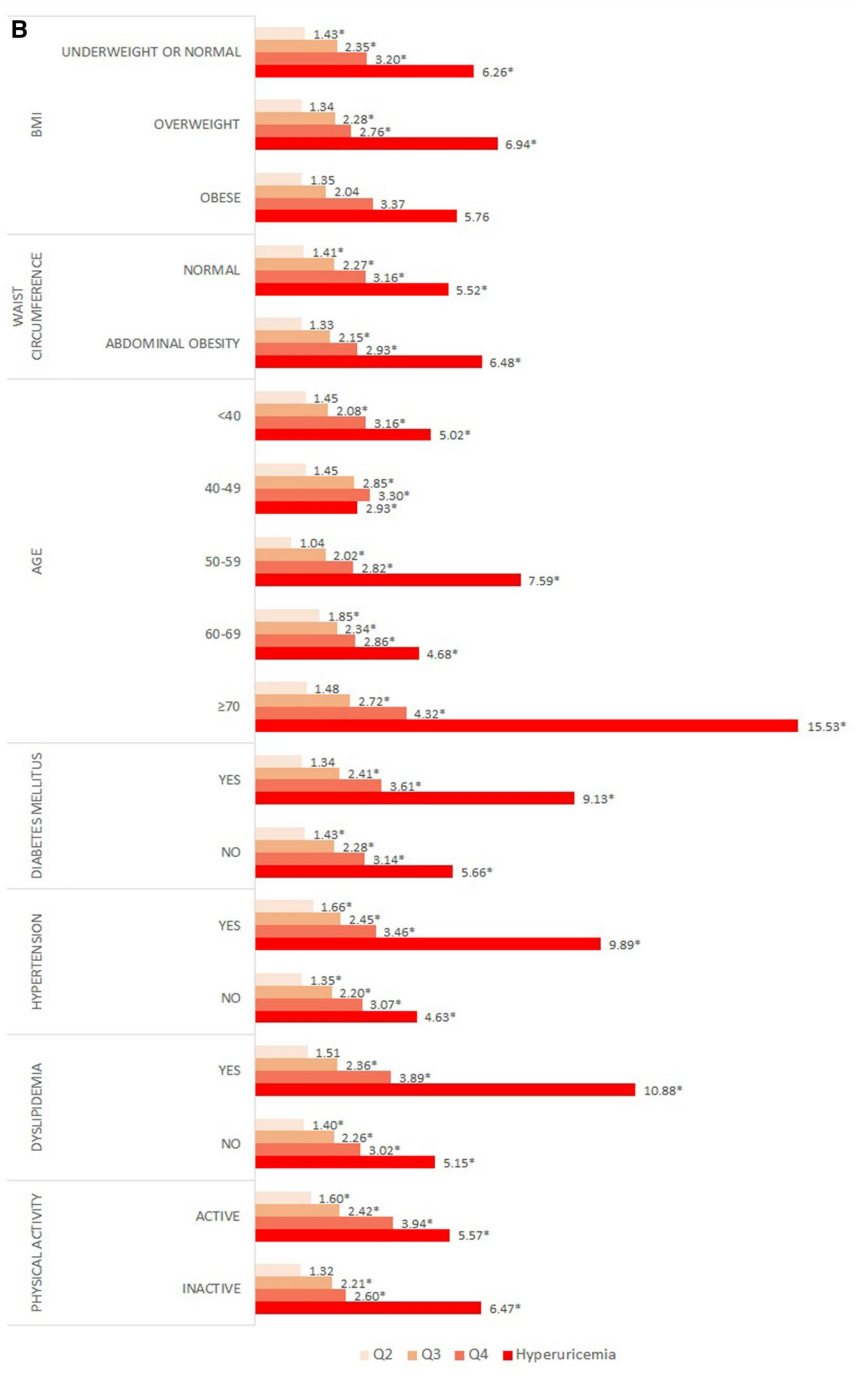
Subgroup analysis presenting odds ratio for decreased kidney function stratified by covariates in (**A**) men, (**B**) women. ^†^Analysis was adjusted for all sociodemographic, economic, health-related factors considered in the study. ^‡^* indicate statistically significant results (*P* < .05).

Fig. 1A and B present the results of the subgroup analysis stratified by gender. For BMI, both genders maintained the dose–response relationship in all BMI categories. Among men, the ORs of the normal or underweight group were significantly higher than those of the other groups. In particular, the OR of the hyperuricemia group was 9.86 times higher than that of normal subjects. In contrast, among women, the OR of the hyperuricemia subjects in the overweight group was 6.94 times higher than that of normal subjects.

In the subgroup analysis according to waist circumference and BMI, a dose–response relationship was observed in both the normal and the abdominal obesity groups. Among women, those who had abdominal obesity and hyperuricemia were 6.48 times more likely than normal controls to show decreased kidney function. The likelihood of decreased kidney function in both men and women increased with advancing age. Men who were previously diagnosed with diabetes or hypertension showed significantly higher ORs than did normal subjects; in particular, men with hyperuricemia were 7.71, and 9.79 times higher than the reference group. Women who were previously diagnosed with diabetes, hypertension, and dyslipidaemia had a dose–response relationship between uric acid levels and decreased kidney function. Particularly, in the hyperuricemia group, ORs were the highest at 9.13, 9.89, and 10.88 times. Moreover, subjects with limited physical activity were more likely to have decreased kidney function.

## Discussion

The purpose of this study was to examine the association between uric acid levels and kidney function in the Korean population using the Korean MDRD formula using representative KNHANES data. We also conducted a subgroup analysis according to BMI, waist circumference, diabetes, hypertension, dyslipidemia, and physical activity, which are factors related to kidney disease.

In this study, we measured the GFR using the MDRD formula for the Korean population to evaluate kidney function. While the direct measurement of GFR is considered to be the most accurate way to detect changes in kidney function, direct measurement requires special skills and involves complex measurements. Therefore, the eGFR is often used in clinical practice. The MDRD formula was derived from studies on the relationship between protein intake and kidney failure progression in patients with CKD in the United States. However, since the MDRD formula was derived mainly from research conducted in populations of European descent, the accuracy of the formula was not verified for other races^26,27^. In 2010, a Korean MDRD formula was developed, allowing for more accurate GFR measurement in Koreans than that with the conventional MDRD formula^19^.

We observed a dose–response relationship between uric acid levels and kidney function in both sexes. Our results indicate that an increasing uric acid level is significantly associated with an increased odds ratio of decreased kidney function. These results are similar to those of earlier studies confirming that high uric acid levels are a risk factor for kidney dysfunction^15,27–29^. Animal studies have also shown that elevated blood uric acid levels can cause kidney damage^26,30^.

Although there is controversy about the association between uric acid levels and CKD, there are several possible mechanisms by which higher uric acid levels could play a role in the development of CKD. According to previous studies, increased levels of uric acid, especially hyperuricemia, can contribute to endothelial dysfunction by inducing antiproliferative effects on the endothelium and inhibiting the production of nitric oxide^31–33^. Over time, damage to the lining of the blood vessels can lead to CKD^34^. A cohort study indicated that early treatment of hyperuricemia could potentially control CKD^35^.

Women had a slightly higher risk of kidney dysfunction than men, which is consistent with the findings of previous studies^20,36,37^. However, other studies have reported that gout and hyperuricemia affect men more commonly^38^. This is possibly related to the female hormone estrogen, which increases uric acid clearance; indeed, premenopausal women have lower uric acid levels than men, and uric acid levels in women start to rise in the fifth decade of life^38–40^. In particular, kidney function was significantly reduced in both men and women in the hyperuricemia group. Patients with CKD often have hyperuricemia^29,41,42^. A recent study revealed that even asymptomatic hyperuricemia that does not develop into gout can affect kidney function unless it is treated with uric acid-lowering drugs^43^.

BMI and waist circumference are known to affect uric acid levels^44^. Our subgroup analysis revealed that women with overweight and abdominal obesity have a more pronounced association with kidney disease. Previous studies have also shown that people with obesity have a higher risk of kidney disease^45^. In addition, uric acid levels and the likelihood of kidney disease development increased with age, as in previous research studies^46^. Diabetes, hypertension, and dyslipidemia are also powerful risk factors for kidney disease^6,34,47^. In addition, physical activity was found to be positively associated with kidney function^48,49^.

This study has several limitations that should be considered when interpreting the results. First, cross-sectional data cannot be used to infer any causal relationships. However, unlike a previous cross-sectional study that utilized hospitalized patients^50^, our study explored the relationship between uric acid level and kidney function using nationally representative samples of South Korea. Further research is still needed to characterize the longitudinal relationship between uric acid levels and kidney diseases in the South Korean population. Second, factors related to health and behavior, including previously diagnosed diseases, were self-reported. Thus, the possibility of recall bias cannot be discarded, despite the efforts of the surveying agency to reduce such bias. Third, although we adjusted for covariates related to uric acid levels and kidney function, the confounding effect cannot be completely excluded because other confounding variables might exist. Particularly, we did not consider the use of uric acid-lowering drugs such as allopurinol and febuxostat. Fourth, additional formulas, such as the Chronic Kidney Disease Epidemiology Collaboration (CKD-EPI) equation, are available for measuring the eGFR. However, this study did not compare the differences in results using different formulas.

Despite these limitations, our research has several strengths. The use of nationally representative data allows our results to be generalized to the general adult population of South Korea. To the best of our knowledge, few studies has been conducted to investigate the association between uric acid levels and kidney function in the Korean population. Thus, this study is meaningful in that it investigated the correlation between uric acid levels and kidney function according to sex. Furthermore, blood samples were collected using standardized laboratory procedures, thus ensuring an accurate estimate of serum creatinine and uric acid levels.

## Conclusion

Our study demonstrated a significant inverse association between high uric acid level and kidney function in the South Korean population. In particular, in the analysis stratified by obesity status, the association was stronger in BMI ≥ 23 group and abdominally obese participants compared with the normal group. Our research shows that uric acid level has a potential role in developing kidney disease. This could help health policymakers and professionals to implement strategies of prevention and intervention for CKD, such as blood screening for patients at a high risk of CKD.

## Supplementary information

Supplementary Table S1.
