# Supplementary material for: Uric acid level and kidney function: a cross-sectional study of the Korean national health and nutrition examination survey (2016–2017)
Source: Sci Rep. 2020 Dec 10;10:21672. doi: 10.1038/s41598-020-77702-x (PMC7730446; doi:10.1038/s41598-020-77702-x)
Supplement: Supplementary file 1 — Supplementary Table S1. [file 41598_2020_77702_MOESM1_ESM.docx]

**Uric Acid Level and Kidney Function: A Cross-sectional Study of the Korean National Health and Nutrition Examination Survey (2016–2017)**

Hye Jin Joo^1,2^: hjjoo22@yuhs.ac

Gyu Ri Kim^2,3^: GYURIKIM@yuhs,ac

Dong-Woo Choi^1,2^: cdw6027@yuhs.ac

Jae Hong Joo^1,2^: jhj3040@yuhs.ac

Eun-Cheol Park, MD, PhD^2,3*^: ecpark@yuhs.ac

^1^Department of Public Health, Graduate School, Yonsei University, Seoul, Republic of Korea

^2^Institute of Health Services Research, Yonsei University, Seoul, Republic of Korea

^3^Department of Preventive Medicine, Yonsei University College of Medicine, Seoul, Republic of Korea

***Corresponding author: Eun-Cheol Park, MD, PhD**

Department of Preventive Medicine and Institute of Health Services Research, Yonsei University College of Medicine

50 Yonsei-ro, Seodaemun-gu, Seoul 03722, Republic of Korea

Tel: +82-2-2228-1862; E-mail: ecpark@yuhs.ac; Fax: +82-2-392-8133

**Supplementary**

| **Supplementary Table S1** Results of subgroup analysis for the association between uric acid level and kidney function according to different factors | | | | | | | | | | | | | | | | | |
| --- | --- | --- | --- | --- | --- | --- | --- | --- | --- | --- | --- | --- | --- | --- | --- | --- | --- |
| **Variables** | **Kidney function: eGFR (mL/min per 1.73m²)^a^** | | | | | | | | | | | | | | | | |
|  | **Q1** | **Q2** | | | | **Q3** | | | | **Q4** | | | | **Hyperuricemia** | | | |
|  | **OR** | **OR** | **95% CI** | | | **OR** | **95% CI** | | | **OR** | **95% CI** | | | **OR** | **95% CI** | | |
| **Men** |  |  |  |  |  |  |  |  |  |  |  |  |  |  |  |  |  |
| **BMI^b^** |  |  |  |  |  |  |  |  |  |  |  |  |  |  |  |  |  |
| Underweight or Normal (<23.0) | 1.00 | 2.21 | (0.87 | - | 5.61) | 2.63 | (1.03 | - | 6.71) | 5.61 | (2.23 | - | 14.06) | 9.86 | (4.18 | - | 23.25) |
| Overweight (23.0-24.9) | 1.00 | 1.68 | (0.72 | - | 3.88) | 1.86 | (0.83 | - | 4.17) | 3.25 | (1.45 | - | 7.28) | 6.30 | (2.81 | - | 14.16) |
| Obese (≥ 25.0) | 1.00 | 1.01 | (0.51 | - | 2.02) | 1.13 | (0.58 | - | 2.19) | 2.00 | (1.03 | - | 3.88) | 3.72 | (1.99 | - | 6.97) |
| **Waist circumference** |  |  |  |  |  |  |  |  |  |  |  |  |  |  |  |  |  |
| Normal | 1.00 | 2.11 | (0.99 | - | 4.47) | 2.44 | (1.19 | - | 5.03) | 4.95 | (2.45 | - | 10.01) | 6.94 | (3.43 | - | 14.05) |
| Abdominal obesity | 1.00 | 1.10 | (0.63 | - | 1.94) | 1.27 | (0.73 | - | 2.21) | 2.15 | (1.24 | - | 3.72) | 4.68 | (2.75 | - | 7.95) |
| **Age** |  |  |  |  |  |  |  |  |  |  |  |  |  |  |  |  |  |
| <40 | 1.00 | 0.45 | (0.17 | - | 1.22) | 0.44 | (0.16 | - | 1.20) | 1.12 | (0.47 | - | 2.68) | 1.72 | (0.73 | - | 4.03) |
| 40-49 | 1.00 | 2.10 | (0.65 | - | 6.85) | 2.31 | (0.74 | - | 7.20) | 4.94 | (1.74 | - | 14.07) | 5.77 | (1.96 | - | 17.00) |
| 50-59 | 1.00 | 1.87 | (0.68 | - | 5.11) | 2.16 | (0.82 | - | 5.66) | 2.64 | (0.99 | - | 7.05) | 5.74 | (2.11 | - | 15.61) |
| 60-69 | 1.00 | 1.98 | (0.87 | - | 4.47) | 3.27 | (1.46 | - | 7.34) | 3.45 | (1.57 | - | 7.59) | 12.24 | (5.58 | - | 26.84) |
| ≥70 | 1.00 | 1.86 | (0.92 | - | 3.77) | 2.13 | (1.07 | - | 4.22) | 4.69 | (2.45 | - | 8.99) | 19.50 | (10.12 | - | 37.57) |
| **Diabetes mellitus** |  |  |  |  |  |  |  |  |  |  |  |  |  |  |  |  |  |
| Yes | 1.00 | 0.89 | (0.35 | - | 2.26) | 2.62 | (1.05 | - | 6.53) | 3.26 | (1.39 | - | 7.62) | 7.71 | (3.16 | - | 18.82) |
| No | 1.00 | 1.57 | (0.92 | - | 2.68) | 1.60 | (0.96 | - | 2.68) | 2.96 | (1.78 | - | 4.92) | 5.31 | (3.28 | - | 8.58) |
| **Hypertension** |  |  |  |  |  |  |  |  |  |  |  |  |  |  |  |  |  |
| Yes | 1.00 | 1.88 | (1.07 | - | 3.33) | 3.09 | (1.78 | - | 5.37) | 3.55 | (2.04 | - | 6.18) | 9.79 | (5.77 | - | 16.60) |
| No | 1.00 | 1.23 | (0.65 | - | 2.32) | 1.19 | (0.65 | - | 2.17) | 2.63 | (1.46 | - | 4.71) | 4.01 | (2.30 | - | 7.00) |
| **Dyslipidemia** |  |  |  |  |  |  |  |  |  |  |  |  |  |  |  |  |  |
| Yes | 1.00 | 1.39 | (0.57 | - | 3.38) | 1.15 | (0.44 | - | 3.04) | 2.29 | (0.93 | - | 5.65) | 4.98 | (2.13 | - | 11.68) |
| No | 1.00 | 1.58 | (0.90 | - | 2.78) | 1.90 | (1.13 | - | 3.21) | 3.36 | (2.01 | - | 5.61) | 5.75 | (3.54 | - | 9.35) |
| **Physical activity** |  |  |  |  |  |  |  |  |  |  |  |  |  |  |  |  |  |
| Active | 1.00 | 1.14 | (0.58 | - | 2.24) | 1.24 | (0.64 | - | 2.40) | 2.42 | (1.28 | - | 4.60) | 3.93 | (2.11 | - | 7.33) |
| Inactive | 1.00 | 1.85 | (1.02 | - | 3.34) | 2.20 | (1.25 | - | 3.86) | 3.70 | (2.12 | - | 6.45) | 7.51 | (4.38 | - | 12.88) |
| **Variables** | **Kidney function: eGFR (mL/min per 1.73m²)^a^** | | | | | | | | | | | | | | | | |
|  | **Q1** | **Q2** | | | | **Q3** | | | | **Q4** | | | | **Hyperuricemia** | | | |
|  | **OR^c^** | **OR** | **95% CI** | | | **OR** | **95% CI** | | | **OR** | **95% CI** | | | **OR** | **95% CI** | | |
| **Women** |  |  |  |  |  |  |  |  |  |  |  |  |  |  |  |  |  |
| **BMI^b^** |  |  |  |  |  |  |  |  |  |  |  |  |  |  |  |  |  |
| Underweight or Normal (< 23.0) | 1.00 | 1.43 | (1.08 | - | 1.88) | 2.35 | (1.79 | - | 3.08) | 3.20 | (2.44 | - | 4.19) | 6.26 | (3.83 | - | 10.21) |
| Overweight (23.0-24.9) | 1.00 | 1.34 | (0.83 | - | 2.17) | 2.28 | (1.44 | - | 3.63) | 2.76 | (1.70 | - | 4.47) | 6.94 | (3.69 | - | 13.04) |
| Obese (≥ 25.0) | 1.00 | 1.35 | (0.79 | - | 2.31) | 2.04 | (1.23 | - | 3.36) | 3.37 | (2.08 | - | 5.47) | 5.76 | (3.49 | - | 9.51) |
| **Waist circumference** |  |  |  |  |  |  |  |  |  |  |  |  |  |  |  |  |  |
| Normal | 1.00 | 1.41 | (1.10 | - | 1.80) | 2.27 | (1.80 | - | 2.86) | 3.16 | (2.49 | - | 4.01) | 5.52 | (3.73 | - | 8.18) |
| Abdominal obesity | 1.00 | 1.33 | (0.78 | - | 2.26) | 2.15 | (1.26 | - | 3.66) | 2.93 | (1.79 | - | 4.79) | 6.48 | (3.83 | - | 10.97) |
| **Age** |  |  |  |  |  |  |  |  |  |  |  |  |  |  |  |  |  |
| <40 | 1.00 | 1.45 | (0.92 | - | 2.29) | 2.08 | (1.36 | - | 3.20) | 3.16 | (2.03 | - | 4.93) | 5.02 | (2.64 | - | 9.54) |
| 40-49 | 1.00 | 1.45 | (0.91 | - | 2.30) | 2.85 | (1.80 | - | 4.49) | 3.30 | (2.12 | - | 5.14) | 2.93 | (1.42 | - | 6.07) |
| 50-59 | 1.00 | 1.04 | (0.65 | - | 1.68) | 2.02 | (1.28 | - | 3.16) | 2.82 | (1.83 | - | 4.35) | 7.59 | (3.97 | - | 14.54) |
| 60-69 | 1.00 | 1.85 | (1.14 | - | 3.02) | 2.34 | (1.45 | - | 3.78) | 2.86 | (1.75 | - | 4.68) | 4.68 | (2.44 | - | 8.99) |
| ≥70 | 1.00 | 1.48 | (0.89 | - | 2.45) | 2.72 | (1.71 | - | 4.33) | 4.32 | (2.76 | - | 6.76) | 15.53 | (7.97 | - | 30.27) |
| **Diabetes mellitus** |  |  |  |  |  |  |  |  |  |  |  |  |  |  |  |  |  |
| Yes | 1.00 | 1.34 | (0.64 | - | 2.83) | 2.41 | (1.25 | - | 4.65) | 3.61 | (1.85 | - | 7.04) | 9.13 | (4.07 | - | 20.50) |
| No | 1.00 | 1.43 | (1.12 | - | 1.82) | 2.28 | (1.83 | - | 2.84) | 3.14 | (2.51 | - | 3.93) | 5.66 | (4.17 | - | 7.68) |
| **Hypertension** |  |  |  |  |  |  |  |  |  |  |  |  |  |  |  |  |  |
| Yes | 1.00 | 1.66 | (1.02 | - | 2.71) | 2.45 | (1.58 | - | 3.79) | 3.46 | (2.26 | - | 5.28) | 9.89 | (5.97 | - | 16.40) |
| No | 1.00 | 1.35 | (1.05 | - | 1.74) | 2.20 | (1.74 | - | 2.77) | 3.07 | (2.43 | - | 3.88) | 4.63 | (3.28 | - | 6.54) |
| **Dyslipidemia** |  |  |  |  |  |  |  |  |  |  |  |  |  |  |  |  |  |
| Yes | 1.00 | 1.51 | (0.89 | - | 2.55) | 2.36 | (1.50 | - | 3.71) | 3.89 | (2.47 | - | 6.11) | 10.88 | (5.68 | - | 20.84) |
| No | 1.00 | 1.40 | (1.10 | - | 1.79) | 2.26 | (1.79 | - | 2.84) | 3.02 | (2.41 | - | 3.80) | 5.15 | (3.75 | - | 7.07) |
| **Physical activity** |  |  |  |  |  |  |  |  |  |  |  |  |  |  |  |  |  |
| Active | 1.00 | 1.60 | (1.15 | - | 2.22) | 2.42 | (1.79 | - | 3.26) | 3.94 | (2.85 | - | 5.46) | 5.57 | (3.57 | - | 8.68) |
| Inactive | 1.00 | 1.32 | (0.98 | - | 1.77) | 2.21 | (1.67 | - | 2.91) | 2.60 | (1.98 | - | 3.41) | 6.47 | (4.36 | - | 9.60) |
| Q1: < 4.80 mg/dL, < 3.70 mg/dL; Q2: 4.80 - 5.49 mg/dL, 3.70 - 4.19 mg/dL; Q3: 5.50 - 6.09 mg/dL, 4.20 - 4.79 mg/dL; Q4: 6.10 - 6.99 mg/dL, 4.80 - 5.99 mg/dL; Hyperuricemia : ≥ 7.00 mg/dL, ≥ 6.00 mg/dL, respectively. | | | | | | | | | | | | | | | | | |
| OR adjusted for all sociodemographic, economic, health-related factors considered in the study | | | | | | | | | | | | | | | | | |
| ªModification of Diet in Renal Disease estimated glomerular filtration rate for Korean population (mL/min per 1.73 m²) = 107.904 x (Creatinine in mg/dL)^-1.009^ x (age)^-0.02^ x 0.667 [if female]. | | | | | | | | | | | | | | | | | |
| ^b^BMI body mass index; Obesity status defined by BMI based on 2018 Clinical Practice Guidelines for Overweight and Obesity in Korea | | | | | | | | | | | | | | | | | |
